# Crystal structure and compressibility of magnesium chloride heptahydrate found under high pressure

**DOI:** 10.1107/S205252062400903X

**Published:** 2024-10-21

**Authors:** Keishiro Yamashita, Kazuki Komatsu, Takanori Hattori, Shinichi Machida, Hiroyuki Kagi

**Affiliations:** a Geochemical Research Center, Graduate School of Science The University of Tokyo Hongo 7-3-1 Bunkyo-ku Tokyo113-0033 Japan; b Institute of Physical Chemistry University of Innsbruck Innrain 52c Innsbruck Tirol6020 Austria; c J-PARC Center Japan Atomic Energy Agency 2-4 Shirakata Tokai, Naka Ibaraki319-1195 Japan; d Neutron Science and Technology Center Comprehensive Research Organization for Science and Society (CROSS) IQBRC Building, 162-1 Shirakata Tokai, Naka Ibaraki319-1106 Japan; University of Erlangen-Nürnberg, Germany

**Keywords:** salt hydrate, high pressure, hydrogen bond, orientational disorder, isothermal compressibility

## Abstract

The odd hydration number has so far been missing in the water-rich magnesium chloride hydrate series (MgCl_2_·*n*H_2_O). In this study, magnesium chloride heptahydrate, MgCl_2_·7H_2_O (or MgCl_2_·7D_2_O), which forms at high pressures above 2 GPa and high temperatures above 300 K, has been identified. Its structure has been determined by a combination of *in*-*situ* single-crystal X-ray diffraction at 2.5 GPa and 298 K and powder neutron diffraction at 3.1 GPa and 300 K. The single-crystal specimen was grown by mixing alcohols to prevent nucleation of undesired crystalline phases. The results show orientational disorder of water molecules, which was also examined using density functional theory calculations. The disorder involves the reconnection of hydrogen bonds, which differs from those in water ice phases and known disordered salt hydrates. Shrinkage by compression occurs mainly in one direction. In the plane perpendicular to this most compressible direction, oxygen and chlorine atoms are in a hexagonal-like arrangement.

## Abstract 2

*In*-*situ* diffraction measurements reveal that magnesium chloride forms a unique high-pressure phase, a heptahydrate, above 2 GPa. The hydrogen-bonding structure appears to contain orientational disorder.

## 1. Introduction

### 1.1. Magnesium chloride hydrates

Magnesium chloride (MgCl_2_) forms various hydrates (MgCl_2_·*n*H_2_O; *n* = 1, 2, 4, 6, 8, and 12) at atmospheric pressures depending on the MgCl_2_:H_2_O ratio and temperature conditions. As seen in MgCl_2_·6H_2_O (Agron & Busing, 1985 ▸), known as a naturally found mineral (bis­chofite), all the hydrates have even hydration numbers except for MgCl_2_·H_2_O [*n* = 1; Sugimoto *et al.* (2007 ▸)]. More specifically, so-called water-rich MgCl_2_ hydrates, in which the magnesium atom is fully coordinated by water molecules forming MgO_6_ octahedra, have only even numbers of interstitial water molecules. The transformations among MgCl_2_ hydrates have been investigated not only for planetary science interests, but also for applications such as seasonal heat storage (Trausel *et al.*, 2014 ▸; Donkers *et al.*, 2017 ▸).

The dehydration process of MgCl_2_·6H_2_O (Sugimoto *et al.*, 2007 ▸) can provide a hint for even-*n* tendency for *n* < 6, such as the local stability of MgO*_m_*Cl_6−*m*_ octahedra. For *n* ≥ 6, chlorine atoms are inevitably excluded from the octahedra. The even number of interstitial water molecules would be the result of the balance of electric charges and geometric strain in the octahedra with two chlorine atoms in the formula unit. Nevertheless, this is not a natural consequence as exemplified by other inorganic salts which form hydrates with an odd-number for *n* such as LiCl·5H_2_O (Sohr *et al.*, 2018 ▸), MgBr_2_·9H_2_O (Hennings *et al.*, 2013 ▸), CaI_2_·7H_2_O (Hennings *et al.*, 2014 ▸), and MgSO_4_·11H_2_O (Peterson & Wang, 2006 ▸).

### 1.2. Salt hydrates under pressure

Pressure expands the compositional and structural varieties of salt hydrates. For example, meridianiite (MgSO_4_·11H_2_O) decomposes into MgSO_4_·9H_2_O by dehydration (Fortes, Fernandez-Alonso, Tucker & Wood, 2017 ▸), potassium chloride (KCl) forms monohydrate [KCl·H_2_O; Yamashita *et al.* (2022 ▸)], and sodium chloride forms hyperhydrates [NaCl·7.5H_2_O and NaCl·13H_2_O; Journaux *et al.* (2023 ▸)] under pressures. Even with the same compositions, salt hydrates such as MgSO_4_·5H_2_O (Wang *et al.*, 2018 ▸) form high-pressure polymorphs like water ice, which also contributes to structural variety. In the case of MgCl_2_, two high-pressure forms are known: MgCl_2_·6H_2_O-II (Yamashita *et al.*, 2019 ▸) and MgCl_2_·10H_2_O (Komatsu *et al.*, 2015 ▸). MgCl_2_·6H_2_O-II has a distorted structure from bis­chofite above 0.6 GPa. MgCl_2_·10H_2_O had been a missing even-*n* hydrate for *n* ≤ 12 but was found to form at 2.1–2.3 GPa. Even in such varieties in hydration numbers, odd-*n* water-rich hydrates have been missing.

### 1.3. Preliminary observation of unknown hydrate

In our previous study on MgCl_2_·10H_2_O, which forms from saline amorphous solution upon heating at ≃2.7 GPa (Komatsu *et al.*, 2015 ▸), some unknown diffraction peaks were observed before the formation of MgCl_2_·10H_2_O. The unidentified peaks did not correspond to any known high-pressure phases of ice or hydrates. Moreover, upon the formation of the unknown peak along with the disappearance of MgCl_2_·10H_2_O, the diffraction intensity of ice VII increased (Fig. S1). Hence, we hypothesized that the unknown phase is a new MgCl_2_ hydrate with a hydration number less than ten in a similar manner to the dehydration of MgSO_4_·11H_2_O into MgSO_4_·9H_2_O (Fortes, Fernandez-Alonso, Tucker & Wood, 2017 ▸). In this study, we identified it as magnesium chloride heptahydrate (MgCl_2_·7H_2_O), a missing odd-*n* hydrate in the MgCl_2_ hydrate family, which will be shown later.

Our preliminary experiments (details in Section S1) suggested that the new phase forms dominantly from the dodecahydrate at 2–4 GPa and ≥320 K. However, its structure could not be determined even for its lattice parameters or the hydration number from the difficulty in indexing the peaks in the powder diffraction pattern even by using a synchrotron source or neutron in our previous studies (Komatsu *et al.*, 2015 ▸). Furthermore, the single-crystal diffraction which is a strong method to determine them was also not straightforward. Single crystals of the high-pressure phase can be directly grown from a solution by cyclic compression or heating (Fabbiani *et al.*, 2007 ▸; Oswald *et al.*, 2008 ▸), but the *pT* region where we can nucleate a seed crystal is limited to be on the intrinsic liquidus of the solution. This *pT* limitation is problematic for obtaining single-crystalline specimens *in situ* in the case of polymorphic materials such as water ice and binary systems such as salt hydrates. Lower-pressure phases compared to the new phase such as ice VI, MgCl_2_·6H_2_O, and MgCl_2_·10H_2_O initially form during compression (Komatsu *et al.*, 2015 ▸; Yamashita *et al.*, 2019 ▸) and the sample space is usually filled by a mixture of polycrystals of various phases, which hampers the growth of single crystals of the desired hydrate and the structural analyses due to the overlaps of diffraction spots.

### 1.4. Strategy of this study

The temperature for the diffraction measurement (298 K) is lower than the melting temperature of ice VII at the required pressure [381 K at 2.5 GPa; Dunaeva *et al.* (2010 ▸)]. Here, we suppressed the nucleation of multi-crystals by expanding the liquid region towards higher pressures (or towards lower temperatures) *via* adding alcohols to initial solutions (Yamashita, Komatsu, Klotz *et al.*, 2022 ▸). The key point of this technique is to match the liquidus of the solution to the stable region of the target phase.

In this study, we investigated the crystal structure of this unknown hydrate using both X-ray and neutron diffraction techniques with computational complementation. We conducted *in*-*situ* single-crystal X-ray diffraction experiments on the sample grown by the alcohol-mixing technique and derived the initial structure model including the chemical composition. Based on the obtained model, the hydrogen positions were determined by powder neutron diffraction. Moreover, its compression behaviours were examined by sequential powder X-ray diffraction measurements using a synchrotron radiation source. Density functional theory (DFT) calculations were also performed to complement the experimental observations. Hereafter, the MgCl_2_ hydrates with hydration number, *n*, will be abbreviated as MC*n*, and their deuterated counterparts are denoted MC*n*d if necessary to distinguish them from the natural isotope compounds, MC*n*h (*e.g*. MC7d for MgCl_2_·7D_2_O and MC7h for MgCl_2_·7H_2_O).

## 2. Experimental

### 2.1. Sample preparation

The single crystal of the unknown MgCl_2_ hydrate was obtained by crystallization at high pressures from a mixture of saturated MgCl_2_ aqueous solution and 4:1 methanol–ethanol (ME). ME is widely used as a pressure-transmitting medium which retains hydro­static conditions up to 10 GPa (Klotz, Chervin, Munsch & Le Marchand, 2009 ▸). A saturated MgCl_2_ aqueous solution was first prepared by dissolving reagent-grade magnesium chloride hexahydrate powder (MgCl_2_·6H_2_O, *M*_r_ 203.30, > 98 wt%, WAKO) into milli-Q water up to the saturated amount (*ca* MgCl_2_: H_2_O = 1:10 in molar ratio at room temperature). This saline solution was mixed with ME in a 2:3 volumetric ratio to accomplish liquid–solid coexistence without other crystalline phases at pressures of approximately 2–4 GPa, where MgCl_2_·7H_2_O stably forms at room temperature. This mixing ratio was determined by the following procedure. First, the mixing ratio was set to 1:1 by volume. If unwanted crystals remained below the targeted pressure, the amount of ME was increased. If no crystals emerged at higher pressures than the limit for the stable operation (≃4 GPa), the ratio of ME was reduced. The optimal ratio was determined by repeating these processes until crystals of the intended phase were grown into a large crystal sufficient for single-crystal diffraction. When ME was not mixed, unwanted crystals of hydrates and high-pressure phases of ice first crystallized at lower pressure.

For neutron diffraction experiments, deuterated MgCl_2_ hexahydrate was prepared by iterative recrystallization with D_2_O as explained in our previous work on MC6 (Yamashita *et al.*, 2019 ▸). The obtained deuterated hydrate was dissolved in D_2_O to an almost saturated concentration (MgCl_2_:D_2_O ≃ 1:11). Non-deuterated solutions with almost the same concentration were used in additional synchrotron X-ray powder diffraction experiments.

### 2.2. Single-crystal X-ray diffraction

Single-crystal X-ray diffraction was carried out *in situ* under pressure. Sample solutions containing the alcohol mixture were loaded into a diamond anvil cell (DAC) made of CuBe alloy equipped with Boehler–Almax-type diamond anvils (Boehler & De Hantsetters, 2004 ▸; Boehler, 2006 ▸) and tungsten carbide (WC) seats for wide accessibility for the incident and scattered X-rays (Komatsu *et al.*, 2011 ▸; Yamashita *et al.*, 2022 ▸). The sample was loaded in the double gasket: the outer gasket was made of 100 µm-thick stainless steel (JIS SUS301) with a 300-µm diameter hole at the centre. An inner gasket made of perfluoro­alk­oxy alkanes (Teflon^TM^ PFA) with 100 µm thickness, 100 µm inner and 300 µm outer diameters, was inserted in the outer gasket hole to position a single crystal at the centre of the sample space. This inner gasket also prevents incident/diffracted X-rays from passing through the metal gasket (Komatsu *et al.*, 2011 ▸). Four cartridge heaters were used to heat the sample to promote the crystal growth (Fig. S2). The sample pressures were estimated by the ruby fluorescence method (Piermarini *et al.*, 1975 ▸; Ragan *et al.*, 1992 ▸).

**Figure 1:**
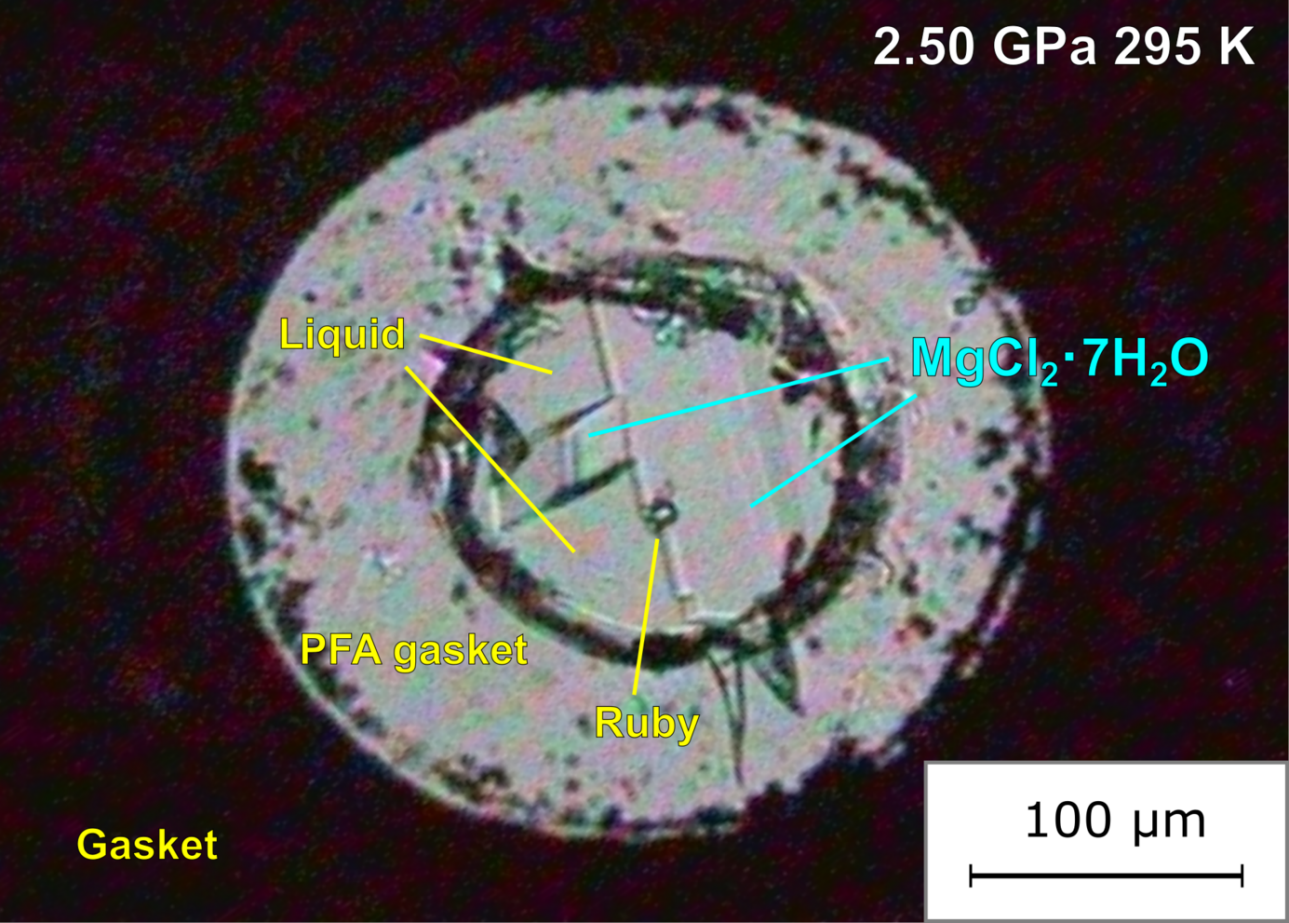
Photograph of single crystals of MgCl_2_·7H_2_O at 2.5 GPa used in the single-crystal X-ray diffraction experiment.

Upon compression at 298 K, no crystals appeared from the sample solution even at 4.5 GPa (Fig. S3). Crystals nucleated after heating to 318 K. The crystallization proceeded slowly and the sample space filled with polycrystals after leaving for one hour. Undesired crystals were dissolved by decompression or heating while compression or cooling enabled the crystals grow. Seed crystals were obtained at ≃1.8 GPa and ≃ 315 K after iterative heating/cooling and compression/decompression, in a similar way to our previous work (Yamashita *et al.*, 2019 ▸). The remaining two crystals (Fig. S3c) were grown by applying pressure up to 2.2 GPa and slowly cooling down to 298 K over a period of two hours (Fig. 1 ▸).

The incident X-ray beam with a wavelength of λ = 0.71075 Å (Mo *K*α, MicroMAX-007, Rigaku) was collimated to a diameter of 300 µm. Diffraction data of MC7h were collected with R-AXIS IV^++^ (Rigaku) diffractometer. The details of the single-crystal X-ray diffraction are summarized in Table 1 ▸.

### 2.3. Powder neutron diffraction

Powder neutron diffraction was measured on beamline BL11 (PLANET) at MLF J-PARC (Hattori *et al.*, 2015 ▸). The saturated MgCl_2_–D_2_O solution was introduced into a pair of encapsulating TiZr gaskets with a tapered aluminium ring (Iizuka *et al.*, 2012 ▸). A pair of alumina-toughened zirconia (ATZ) anvils (Komatsu *et al.*, 2014 ▸) were used instead of stiffer WC anvils to reduce the neutron attenuation by the anvils. It is because MC7 has a long *c*-length and diffraction intensities at large *d* value (*i.e.* long wavelength neutrons) are strongly attenuated by WC anvils. Pressure and temperature were controlled using the MITO system (Komatsu *et al.*, 2013 ▸). No pressure marker, such as Pb or Au, was loaded in the sample space to avoid contamination of diffraction patterns. Instead, the sample pressures were estimated from the lattice parameter of coexisting ice VII (Klotz *et al.*, 2017 ▸).

The sample was compressed up to approximately 3 GPa at 300 K and heated to 353 K. After the completion of the transition (≃4 h), the diffraction pattern was collected at 300 K and 3.1 GPa for 12 h at the proton beam power of approximately 500 kW. In the data collection, we employed the double frame mode (12.5 Hz) to extend the measurable *d*-range up to 8.4 Å. Experimental details are given in Table 1 ▸.

### 2.4. Sequential powder diffraction measurements with synchrotron X-ray source

Sequential powder X-ray diffraction measurements were performed at the BL-18C beamline of the Photon Factory (PF), High Energy Accelerator Research Organization (KEK). The sample crystals were prepared in a similar way to the neutron experiments except for the sample solution (MgCl_2_:H_2_O = 1:11) and the high-pressure device (the Boehler–Almax-type DAC equipped with culet diameters of 600 µm). A stainless steel gasket (JIS SUS304) with an initial thickness of 100 µm was used. A hole with a diameter of 300 µm was drilled as a sample space.

Monochromatic X-ray beam was collimated to a diameter of 100 µm. Diffraction data were collected for 300 s by an angular-dispersive method using a Rad-icon 2022 CMOS detector (Teledyne Rad-icon Imaging Corporation). The diffractometer parameters were calibrated using CeO_2_ as a standard, giving the incident beam wavelength of λ = 0.61940 (13) Å. The collected two-dimensional diffraction patterns were reduced into one-dimensional profiles using *IPAnalyzer* (Seto *et al.*, 2010 ▸) software.

Diffraction patterns were collected at 298 K upon compression from 2.5 to 4.8 GPa and decompression to 2.7 GPa with intervals of 0.1–0.3 GPa. At the highest pressure, the sample was heated at 318 K for one hour to remove the strain in the sample space. The transparency in the sample space changed and diffraction peaks started to broaden at 3 GPa, suggesting that the sample fully solidified above 3 GPa. The diffraction profiles were collected from the mixture of the hydrate and ice VII and the pressure was determined from the equation of state of the coexisting ice VII (Klotz *et al.*, 2017 ▸).

### 2.5. Data reduction and structural analysis

Peak picking and indexing for single-crystal X-ray diffraction were conducted using the *CrystalClear* (Rigaku, 2015 ▸) software. Absorption by diamond anvils was corrected manually in the listed reflections [see Section S2 in Yamashita *et al.* (2022 ▸)]. The initial structure of MC7 was derived using the direct method in *SIR2014* (Giacovazzo, 1980 ▸), and the structure parameters such as atomic positions and displacement parameters were refined by *SHELXL* (Sheldrick, 2015 ▸). The hydration number was also determined from the derived structure. Hydrogen atoms were not included considering the small scattering factor of hydrogen and relatively large errors of the scattering intensities due to the high-pressure cell. Some reflections with large variations caused by the high-pressure cell were omitted as long as the goodness of fit was not less than one. The derived structure model without hydrogen sites was used as an initial structure model in the subsequent analysis of the powder neutron diffraction data.

The collected powder neutron diffraction patterns were reduced into one-dimensional profiles and normalized using data taken for a vanadium pellet and empty cell loaded in the MITO system. The structure determination of MC7d including the deuterium positions was conducted by the difference Fourier method based on the Rietveld analysis using the *GSAS* programme (Larson & Von Dreele, 2004 ▸) with *EXPGUI* (Toby, 2001 ▸). The final results of the Rietveld analysis account for the 14 D-atom sites. In the refinement, atomic displacement parameters of oxygen and deuterium were constrained to be the same among the same atomic species to reduce the number of parameters. Each D-atom site was refined as a mixture of D and H atoms considering the impossibility of deuteration. Their fractions were constrained to be the same among all the D/H-atom sites and the sums of their fractions at the same site fixed to one. The atomic distances of Mg—O and O—D/H were restrained to 2.08 (1) and 0.96 (1) Å, respectively. The correction for the preferred orientation was applied in the Rietveld analyses using the March–Dollase function (March, 1932 ▸; Dollase, 1986 ▸), but this did not affect the refinement results significantly except for a slight improvement of *R* factors.

For sequential X-ray diffraction, the lattice parameters of the hydrate and ice VII were derived by Rietveld analysis using the *GSAS* programme (Larson & Von Dreele, 2004 ▸) with *EXPGUI* (Toby, 2001 ▸). Considering the small atomic scattering factor of hydrogen, structure models without hydrogen atoms are used.

### 2.6. DFT calculations

To complement the powder neutron diffraction analysis, we performed the structure optimization of MC7 with density function theory (DFT) calculations (Hohenberg & Kohn, 1964 ▸; Kohn & Sham, 1965 ▸) using *Quantum Espresso* (Giannozzi *et al.*, 2009 ▸). We used Perdew–Burke–Ernzerhof (so-called PBE) type non-empirical exchange-correlation functions (Perdew *et al.*, 1996 ▸). The pseudopotentials were derived using projector-augmented wave approximation (Kresse & Joubert, 1999 ▸). The dispersion effects were taken into account using the exchange-hole dipole moment (XDM) method, which calculates coefficients for the polynomial of DFT-D dispersion energy (Grimme *et al.*, 2010 ▸) from the exchange-hole dipole moment calculated from the simulated electron wavefunction (Becke & Johnson, 2005 ▸, 2007 ▸). XDM damping function parameters were taken from Roza & DiLabio (2017 ▸) and Otero-De-La-Roza & Johnson (2020 ▸). The formation energy of MC7 was calculated within a unit cell with a kinetic energy cutoff of 2040 eV for wavefunction and a Brillouin zone *k*-mesh of 4 × 4 × 1 (≃ 0.04 Å^−1^ reciprocal lattice spacing) in a cell setting of *P*2_1_/*n*.

The structure model from neutron diffraction was first used as the initial structure except for the use of protium (^1^H) instead of deuterium. These structures were optimized while the unit-cell parameters were fixed to be the experimental values of neutron diffraction at 3.1 GPa and 300 K. The relaxed structure and the neutron data showed some mismatches in hydrogen/deuterium sites. Then, we picked up some additional candidates for hydrogen sites around O1, O6 and O7 based on the difference Fourier map derived from neutron diffraction data and the DFT-optimized structure. By selecting some of these sites, we modelled 27 configurations with different orientations of water molecules (Table S1) and compared their energies based on the same structure optimizations. The structural parameters were optimized using Broyden–Fletcher–Goldfarb–Shanno quasi-Newtonian methods under pressure.

## 3. Results and discussion

### 3.1. Alcohol addition for selective single-crystal growth

As shown in Fig. 1 ▸, two single crystals of MC7h were obtained at 2.5 GPa and 298 K from the alcohol-mixed solution without the coexistence of other crystalline phases. Without alcohol, MgCl_2_ aqueous solution can crystallize into mainly four candidates (ice VI, ice VII, MC6-II and MC10) other than MC7 at pressures up to 4 GPa at 298 K. The alcohol addition decreases the freezing point of the solution, which also causes the increase in crystallization pressure because the melting point increases with pressure. The alcohol addition suppressed the crystallization which can interfere with the structure analysis.

From a technical aspect, the crystallization behaviours of the sample solution during compression varied among individual runs. For example, some runs failed due to ice VII covering the sample space or no crystallization below 4 GPa, the controllable pressure regime for single-crystal diffraction measurement.

### 3.2. Crystal structure of MgCl_2_·7H_2_O (MgCl_2_·7D_2_O)

**Figure 2:**
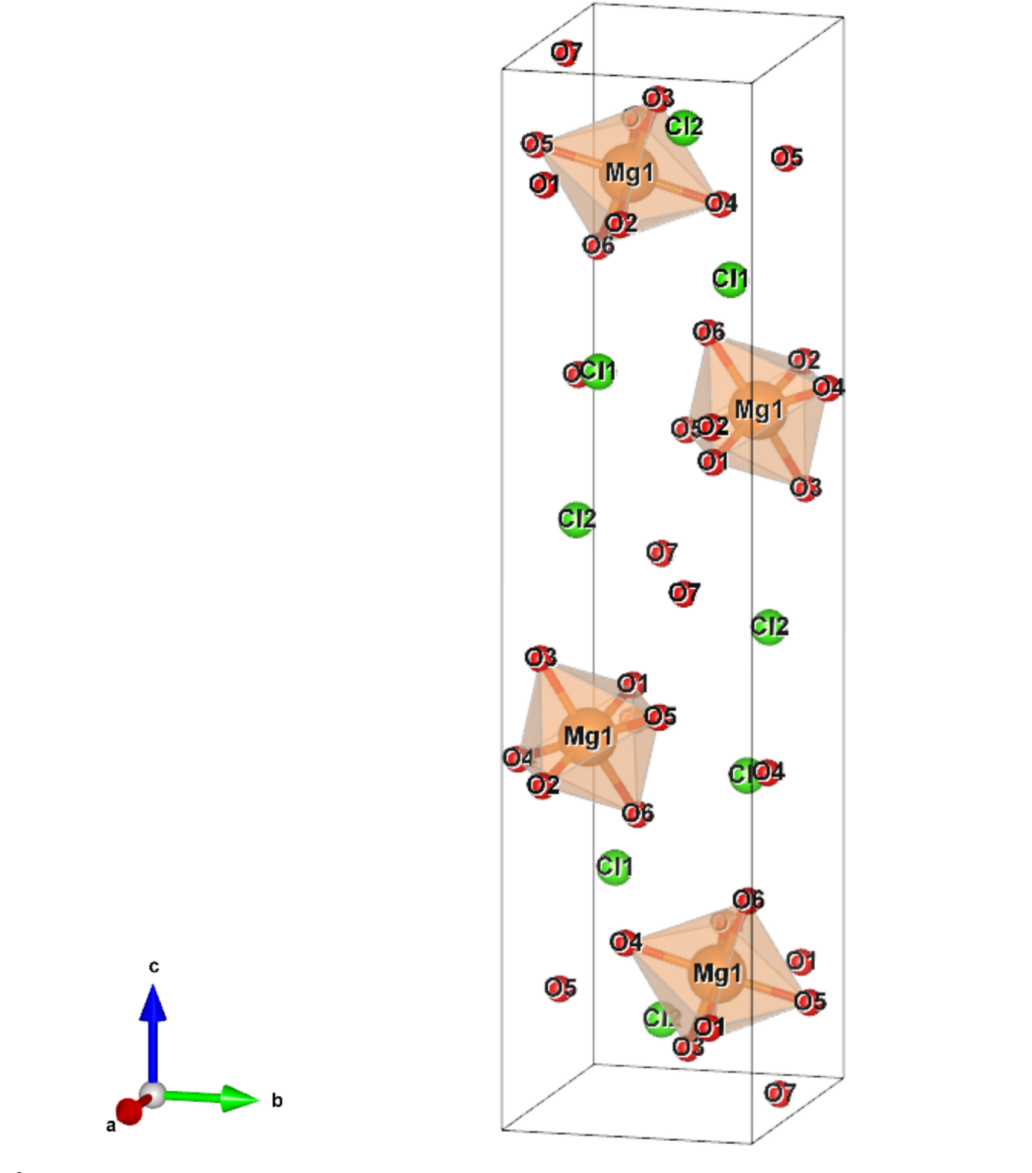
Crystal structure of MgCl_2_·7H_2_O determined in this study. H atoms which connect to O atoms are not shown for clarity.

Table 1 ▸ summarizes the determined lattice parameters of the new hydrate. From the single-crystal X-ray diffraction data, we assigned a monoclinic unit cell with a space group of *P*2_1_/*n* from a systematic absence of *h*0*l* (*h* + *l* ≠ 2*n*) and 0*k*0 (*k* ≠ 2*n*) diffraction peaks. We selected the *P*2_1_/*n* setting (Fig. 2 ▸) instead of *P*2_1_/*c* to avoid unfamiliarly large β ≃ 165°. In this cell setting, MC7 has a large *c* axis (≃23 Å) compared with other axes (*a* ≃ 6.2 Å, *b* ≃ 5.6 Å).

MC7 consists of Mg(H_2_O)_6_ octahedra [Mg(D_2_O)_6_ for MC7d], Cl atoms and interstitial water molecules (Fig. 2 ▸) like other water-rich MgCl_2_ hydrates (Komatsu *et al.*, 2015 ▸; Hennings *et al.*, 2013 ▸), but its hydration number is odd. This hydration number is also supported stoichiometrically by the Rietveld analysis of the powder neutron diffraction data, resulting in the molar ratio between the hydrate and ice VII of 1:4.42 (6), in close agreement with the ratio of 1:4 expected from the starting solution of MgCl_2_:D_2_O ≃ 1:11.

**Figure 3:**
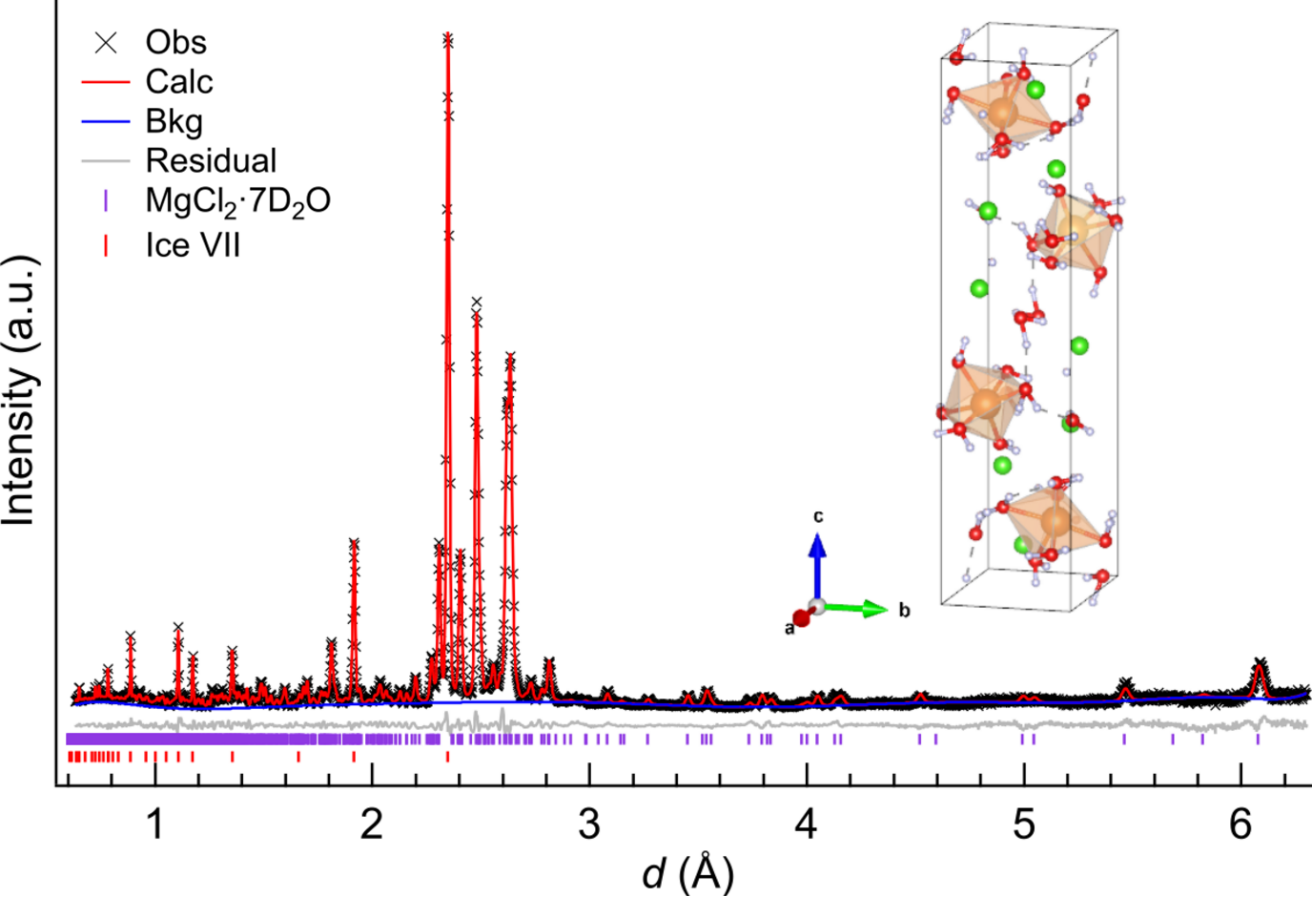
Powder neutron diffraction pattern of MgCl_2_·7D_2_O at 3.1 GPa and 300 K. The inset shows the refined crystal structure of MgCl_2_·7D_2_O. Orange, green, red and pale-blue balls represent magnesium, chlorine, oxygen and deuterium atoms, respectively.

Fig. 3 ▸ shows the powder neutron diffraction profile of MC7d and refined crystal structure. Most of the deuterium positions were determined by the difference Fourier map, but some molecular geometries converged into suspicious ones when the ordered structure model was employed. It was seen in the peculiar hydrogen bonds between adjacent water molecules coordinating on the same magnesium atom (O4—D4*B*⋯O6), or very small intramolecular D—O—D angle [∠D7*A*—O7—D7*B* = 68 (3)°]. These are considered to relate to the inadequate accuracy of neutron diffraction patterns since the monoclinic symmetry with the long-*c* axis induces serious overlap of the Bragg peaks. This problem is compensated by DFT calculations.

### 3.3. Molecular configurations from DFT calculation

**Figure 4:**
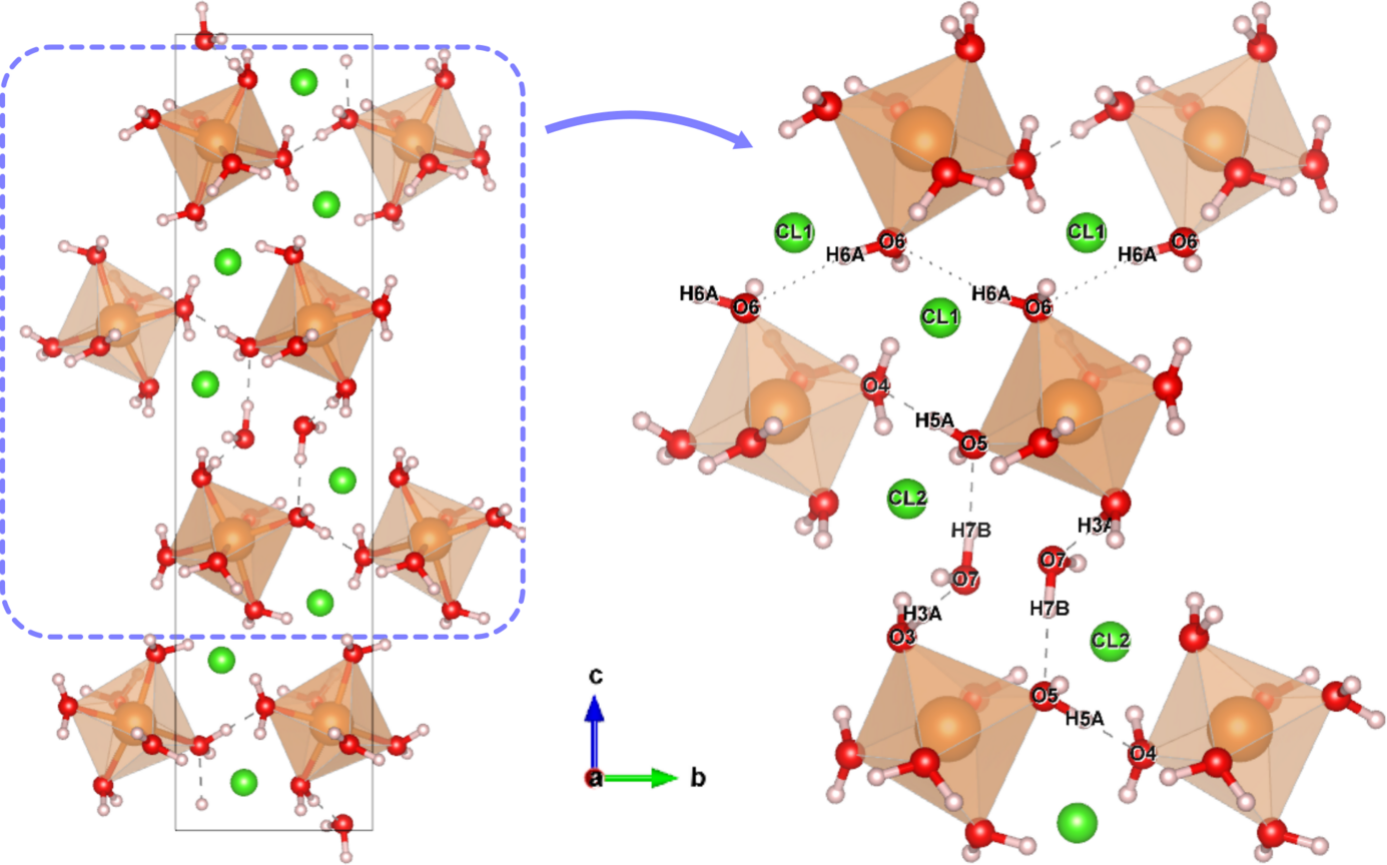
Structure of MgCl_2_·7H_2_O in configuration 1 optimized by DFT calculation. The close-up view of the hydrogen-bonded chain formed by water molecules is shown in the right panel. The hydrogen bonds with *d*(H⋯O) shorter than 2 Å are described by dashed lines. Interatomic H6*A* and O6 bond with *d*(H⋯O) = 2.16 Å are shown by dotted lines.

The DFT-optimized model (Fig. 4 ▸) shows consistent structures with the neutron diffraction result (Fig. 3 ▸). For example, the D2*B* (H2*B* for DFT optimization) forms bifurcated hydrogen bonds to two Cl1. Here, the bifurcation is judged by the simple rule based on the planarity of the four atom sites (Parthasarathy, 1969 ▸). The sum of the three angles of the corresponding atom pairs is 360 (3)°, indicating the four atoms are almost on the same plane. Bifurcated hydrogen bonds are more common in high-pressure phases such as MC10 (Komatsu *et al.*, 2015 ▸). Such a more tightly packed arrangement would be preferred under pressure, similar to the preference for the B2 structure of anhydrous NaCl with a coordination number of eight above 2 GPa instead of six in the B1 phase at ambient pressure.

Mg(H_2_O)_6_ octahedra are connected *via* hydrogen bonds. In contrast to some other hydrates such as MC8 and MC12 (Hennings *et al.*, 2013 ▸), the coordinating water molecules accept hydrogen bonds from other water molecules, forming hydrogen-bond chains of O3—O3*A*⋯O7—O7*B*⋯O5—D5*A*⋯O4, like short helices along the *c* axis, terminated by hydrogen bonds with Cl atoms. Hydrogen bonds from interstitial water to coordinated water molecules are not common in water-rich hydrates but are seen in MC10 (Komatsu *et al.*, 2015 ▸). One large difference from MC10 is the direct bridging between adjacent Mg(H_2_O)_6_ octahedra *via* hydrogen bonds (O5—H5*A*⋯O4; Fig. 4 ▸). In another direction along the *b* axis, O6—H6*A*⋯O6—H6*A*… linkage may form the zigzag chain (Fig. 4 ▸) accepting the long connection of *d*(D6*A*⋯O6) = 2.37 (3) Å from neutron diffraction or 2.18 Å from the DFT optimization. The inter­atomic distances are much longer than ordinary hydrogen-bonded D⋯O distances (1.8–2.0 Å in ice VII and VIII) but are still shorter than the sum of van der Waals radii of H and O [2.45–2.72 Å; Batsanov (2001 ▸)]. Such direct hydrogen bonds between cation-centred octahedra are observed in a limited case for salt hydrates [*e.g*. a high-pressure phase of MgSO_4_·5H_2_O (Wang *et al.*, 2018 ▸)]. In the case of MC10, Mg(H_2_O)_6_ octahedra are surrounded by interstitial water molecules and chlorine, so such direct connections are not seen. Hence, at higher pressures, the volume decrease is considered to compensate for the structural distortion which is unfavoured at lower pressures.

In contrast, the DFT-optimized model contains some differences from the neutron diffraction results such as the absence of the suspicious molecular geometries. The H4*B* site orients towards Cl1 rather than O6 in the same Mg-centred octahedra. The ∠H7*A*—O7—H7*B* angle is 104.5°, consistent with the known molecular geometry (∠D—O—D = 102–107°) for ice VII (Yamashita, Komatsu, Klotz *et al.*, 2022 ▸) and VIII (Kuhs *et al.*, 1984 ▸; Jorgensen *et al.*, 1984 ▸). At the same time, some other sites also differ from those refined from the neutron diffraction, *e.g*. in DFT-optimized structure, H1*A* and H1*B* orient more straightly to O2 [(O1—H1*A*⋯O2 = 166.8°) and Cl2 (∠O1—H1*B*⋯Cl2 = 172.8°), respectively, in contrast to the neutron diffraction result [∠O1—D1*A*⋯O2 = 132.2 (18)°; ∠O1—D1*B*⋯Cl2 = 126.3 (19)°].

However, this optimized model still does not sufficiently reproduce the observed diffraction pattern. Structure refinements starting from this optimized model become unstable and converge into unrealistic molecular geometries. The difference Fourier map shows residuals at several positions around oxygen in other directions implying the existence of other deuterium sites forming hydrogen bonds towards chlorine or other water molecules. In particular, residuals around the interstitial water molecules, O7, are the most prominent (Fig. S4). Such residuals indicate potential orientational disorders of water molecules in MC7d. The addition of new deuterium sites on the positions of the residuals in the structure model slightly improved the *R* factors, but further refinement became unstable, and the molecular structures are still far from reasonable geometry. Due to the limitation of data quality available from the powder neutron diffraction pattern, we compared some configurations further by DFT calculations.

The prospected disorder includes the new hydrogen bonds between water molecules and chlorine. Orientational disorders of water molecules have been investigated for decades for hydrogen-bonded crystals, mostly ice polymorphs [*e.g*. Komatsu (2022 ▸)]. In such systems, the orientational disorder can be modelled analytically [*e.g*. McDonald *et al.* (1998 ▸); Kuo *et al.* (2001 ▸)] because only directions of hydrogen bonds vary among configurations while the network framework is retained. The disorder of molecular orientations was also found recently in a salt hydrate, NaCl·13H_2_O, a unique high-pressure form (Yamashita *et al.*, 2023 ▸). Its hydrogen-bond network is interpreted as the derivative from that of ice VI structure and the orientational disorder can be also explained by directionality in a graph representation. On the other hand, the hydrogen-bond chains in MC7 are not completely connected throughout the crystal in contrast to water ice phases (Komatsu, 2022 ▸) and NaCl·13H_2_O (Yamashita *et al.*, 2023 ▸). This means that the disorder in MC7 with the reformation of the hydrogen-bond network is more arbitrary in the network topology. Hence, it is not straightforward to construct the possible molecular configurations in MC7 by the graph-based approach. Then, we investigated the difference in candidate structures by picking up 27 (= 3 × 3 × 3) possible configurations (listed in Table S1) for initial structures with different hydrogen sites around O1, O6, and O7 at which some residuals can be seen in the difference Fourier maps. After the structural optimization, configurations remain distinct from each other.

**Figure 5:**
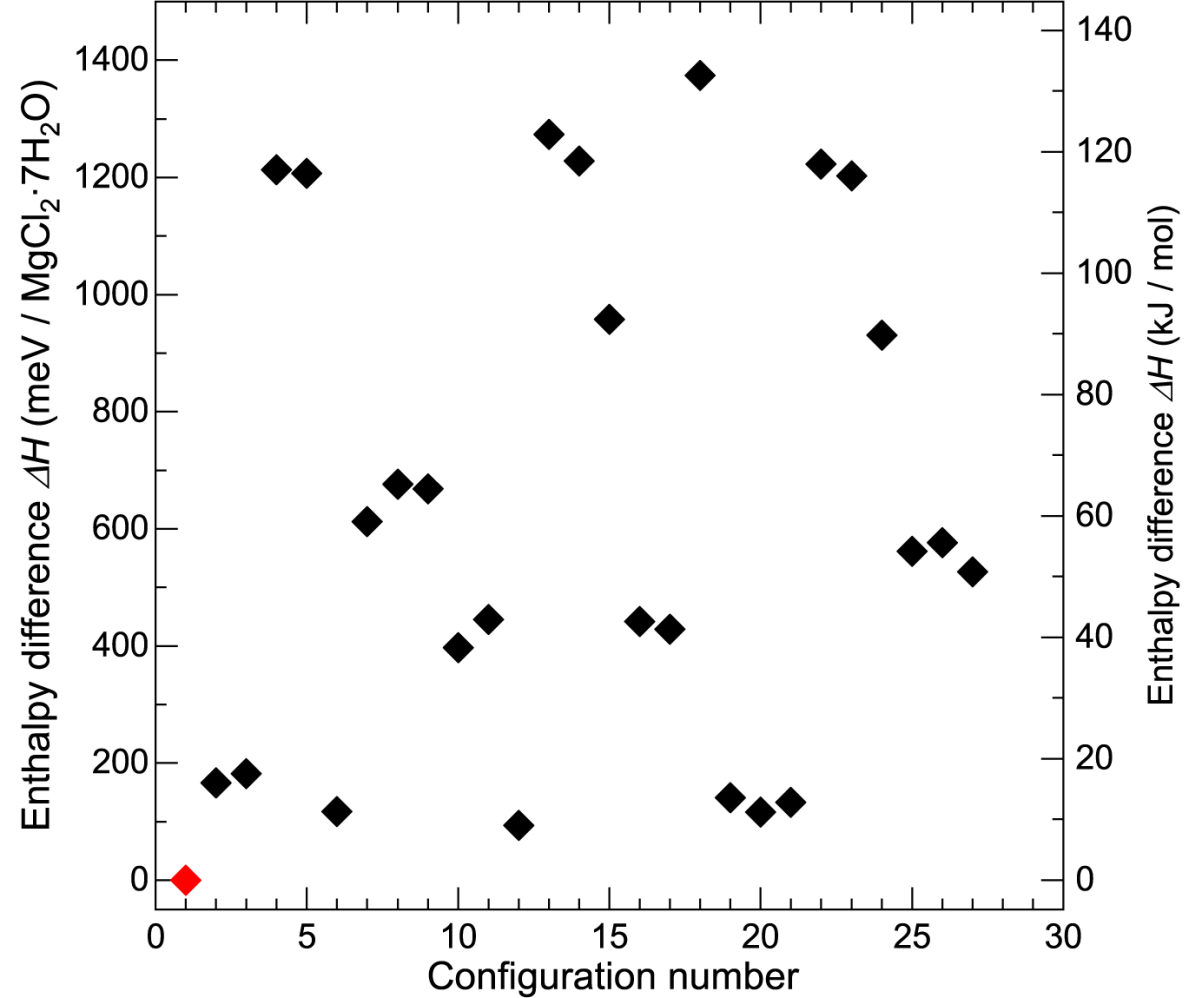
Enthalpies for 27 candidate configurations compared to that of configuration 1 calculated from the DFT structure optimizations. The configuration numbers correspond to those defined in Table S1. The enthalpy for configuration 1 is drawn with a red symbol.

Fig. 5 ▸ summarizes the calculated enthalpies (*H* = *E* + *pV*) where internal energy *E* and the pressure *p* are derived from the calculations with the unit-cell parameters fixed as the experimental values from the neutron diffraction (*V* = 197.765 Å^3^/MgCl_2_·7H_2_O). Configuration 1 (the same structure shown in Fig. 4 ▸) has the lowest enthalpy and the differences from those for some others are within 150 meV/MgCl_2_·7H_2_O. The most unfavoured configuration in the 27 candidates has an enthalpy 1370 meV/MgCl_2_·7H_2_O higher than that of configuration 1. Such differences are rather huge compared to the cases of ice phases [*e.g.* 100 meV/10H_2_O for all configurations of ice VI (Komatsu *et al.*, 2016 ▸)] and NaCl·13H_2_O phases [35 meV/NaCl·13H_2_O for all configurations (Yamashita *et al.*, 2023 ▸)]. The large difference in enthalpy suggests that some configurations are unlikely to occur even if the orientational disorder exists in MC7. That would be the reason why the structure model from neutron diffraction data is rather close to configuration 1.

To understand the possible orientational disorder in MC7 from another aspect, it should be noted that the formation of MC7 did not proceed sufficiently at ≃300 K and ≃3 GPa as seen in the crystallization from amorphous solution [See Fig. 1 ▸ in Komatsu *et al.* (2015 ▸)]. The diffraction peaks from MC7 [referred to as unknown in Komatsu *et al.* (2015 ▸)] remained broad for hours unless heated or decompressed. Such a tendency indicates that the structural rearrangements are kinetically hindered. This is consistent with the idea that molecular reorientations are locked by coexisting ionic species, as seen in salty ice [*e.g*. NH_4_F- and LiCl-doped ice VII (Salzmann *et al.*, 2019 ▸; Klotz, Bove *et al.*, 2009 ▸)] Classical molecular dynamics simulation suggested the locked reorientational dynamics of water molecules in LiCl-doped ice VII can be unlocked by heating from 300 to 450–500 K (Klotz, Bove *et al.*, 2009 ▸). In the same manner, the structure derived from the neutron diffraction data at 300 K may reflect a kinetically frozen state of MC7 (called orientational glass): its molecular configuration would retain the orientational disordered at higher temperatures.

### 3.4. Isothermal compressibility of MgCl_2_·7H_2_O

**Figure 6:**
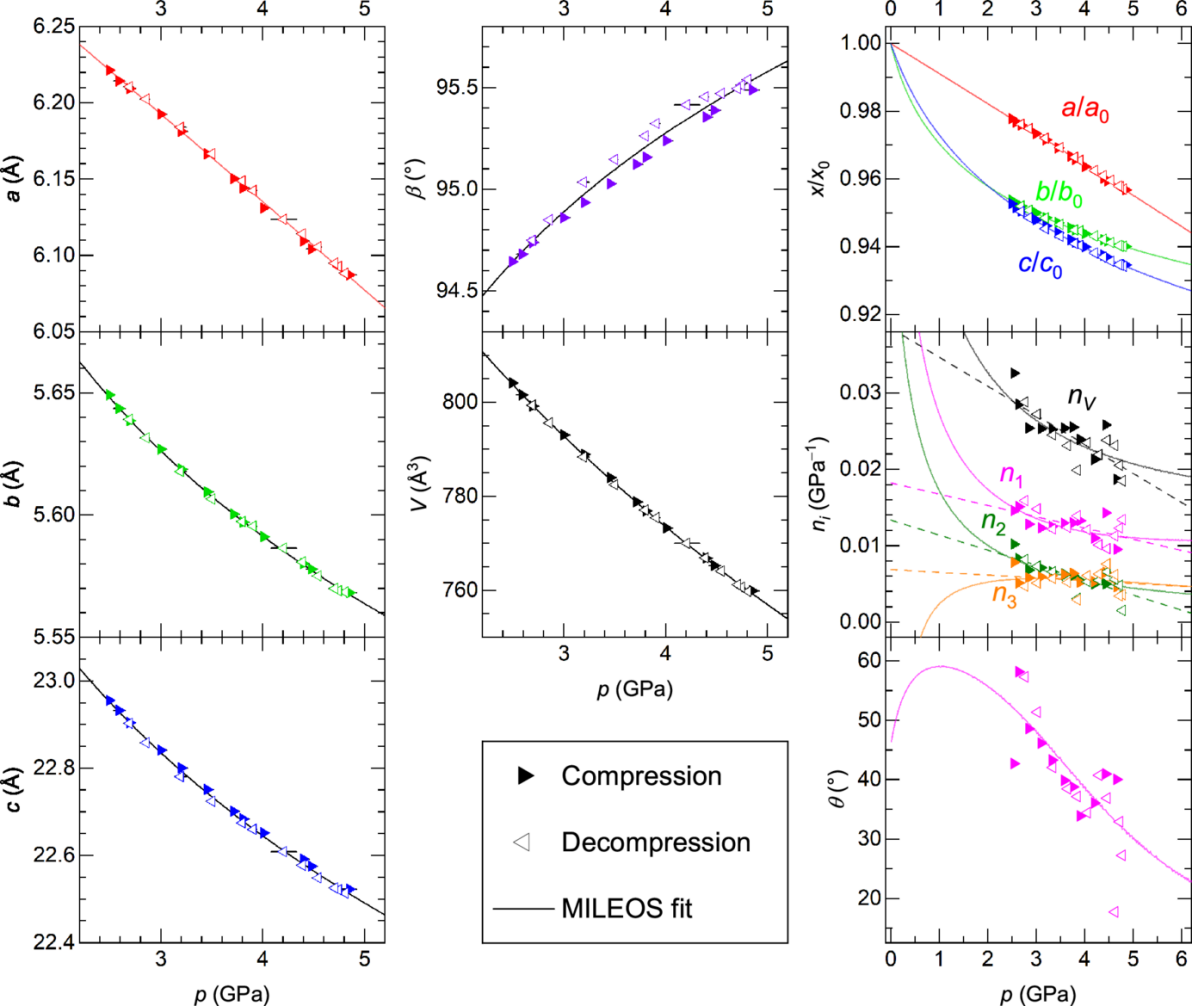
Isothermal compressibility of MgCl_2_·7H_2_O at 298 K obtained from *in*-*situ* powder X-ray diffraction. The sample pressure (*p*) was derived from the equation of state (EoS) of ice VII (Klotz *et al.*, 2017 ▸). MILEOS fitting curves (solid lines) are derived from data from both compression and decompression. Linear compressibilities along principal axes (*n_i_*, where *i* = 1, 2, 3) as well as their sum as the bulk compressibility (*n_V_*) and inclination angle of β_1_ against the *a* axis (θ) derived from the derivatives of the fitted MILEOS curves of the lattice parameters are also described in the solid curves. The linear fits of the compressibilities are also shown in the dashed curves.

Fig. 6 ▸ summarizes the compression behaviours of MC7h derived from the synchrotron powder X-ray diffraction data at 2.5–4.8 GPa and 298 K. For a convenient description, the pressure-dependence of the unit-cell volume is parameterized using a Murnaghan integrated linear equation of state (MILEOS; Murnaghan, 1944 ▸)
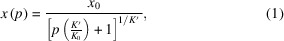
where *K*_0_ is the isothermal bulk modulus at 0 GPa and *K*′ is the first pressure derivative of the bulk modulus (∂*K*_0_/∂*p*). Note that MC7 does not perfectly satisfy the prerequisites for the equation but this function is selected for practical reasons to parametrize the lattice parameters numerically. The lattice parameters were also fitted using the same equation as summarized in Table 2 ▸. The lattice parameters show good agreements between compression and decompression. An extrapolation of the *pV* plot gives *V*_0_ = 879 (4) Å^3^ at 0 GPa.

MC7 is less compressible along the *a* axis (Fig. 6 ▸ and Table 2 ▸). However, the compressibilities along crystallographic axes do not well represent the compression behaviour of the crystal because they are not principal axes of compression. As shown in previous studies on MgSO_4_ hydrates (Fortes, Fernandez-Alonso, Tucker & Wood, 2017 ▸; Fortes *et al.*, 2017 ▸), three directional compressibilities in orthogonal basis are suitable to describe the elastic properties. The elastic strain for a monoclinic structure with α = γ = 90° can be described in a symmetrical second-rank tensor of the form
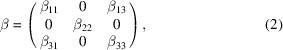
with unit strain components β_*ij*_ corresponding to compressibility coefficients obtained by the methods described in Schlenker *et al.* (1978 ▸) and Hazen & Finger (1982 ▸). The magnitudes and directions (*n*_1_, *n*_2_, *n*_3_) of the principal components can be derived as the eigenvalues and eigenvectors of the matrix. Table 3 ▸ summarizes linear fits for the compressibility of the principal components.

For the monoclinic structure, one of the principal compression axes (*n*_2_) is identical to the *b* axis and the others (*n*_1_ and *n*_3_) are in the *ac* plane. The most compressible direction (*n*_1_) is one of the latter two, and hence the changes in the most compressible direction can be represented by the angle (θ) between *n*_1_ and the crystallographic *a* axis (Fig. 6 ▸). *n*_1_ is approximately along [501] direction and gradually orients towards *a* axis at higher pressures. The compressibility along *n*_1_ is twice larger than the others (*n*_2_ and *n*_3_).

The volume compressibility at 2–5 GPa (0.02–0.03 GPa^−1^) is comparable with those of anhydrous salts, *e.g*. 0.02–0.04 GPa^−1^ for β-MgCl_2_ [calculated from Stavrou *et al.* (2016 ▸)], 0.02–0.03 GPa^−1^ for B1 phase of NaCl [calculated from Matsui *et al.* (2012 ▸)], and slightly smaller than those of ice VII [0.03–0.04 GPa^−1^; calculated from Klotz *et al.* (2017 ▸)]. Thus, overall compression behaviours appear closer to anhydrous salt rather than water ice. On the other hand, the compressibility along the most compressible axis, *n*_1_ of MC7 is larger than the linear compressibilities of such simple systems while the other directional compressibilities (*n*_2_ and *n*_3_) are smaller. Such a large compressibility in a specific direction resembles the compression behaviour of MgSO_4_·11D_2_O (Fortes, Fernandez-Alonso, Tucker & Wood, 2017 ▸).

**Figure 7:**
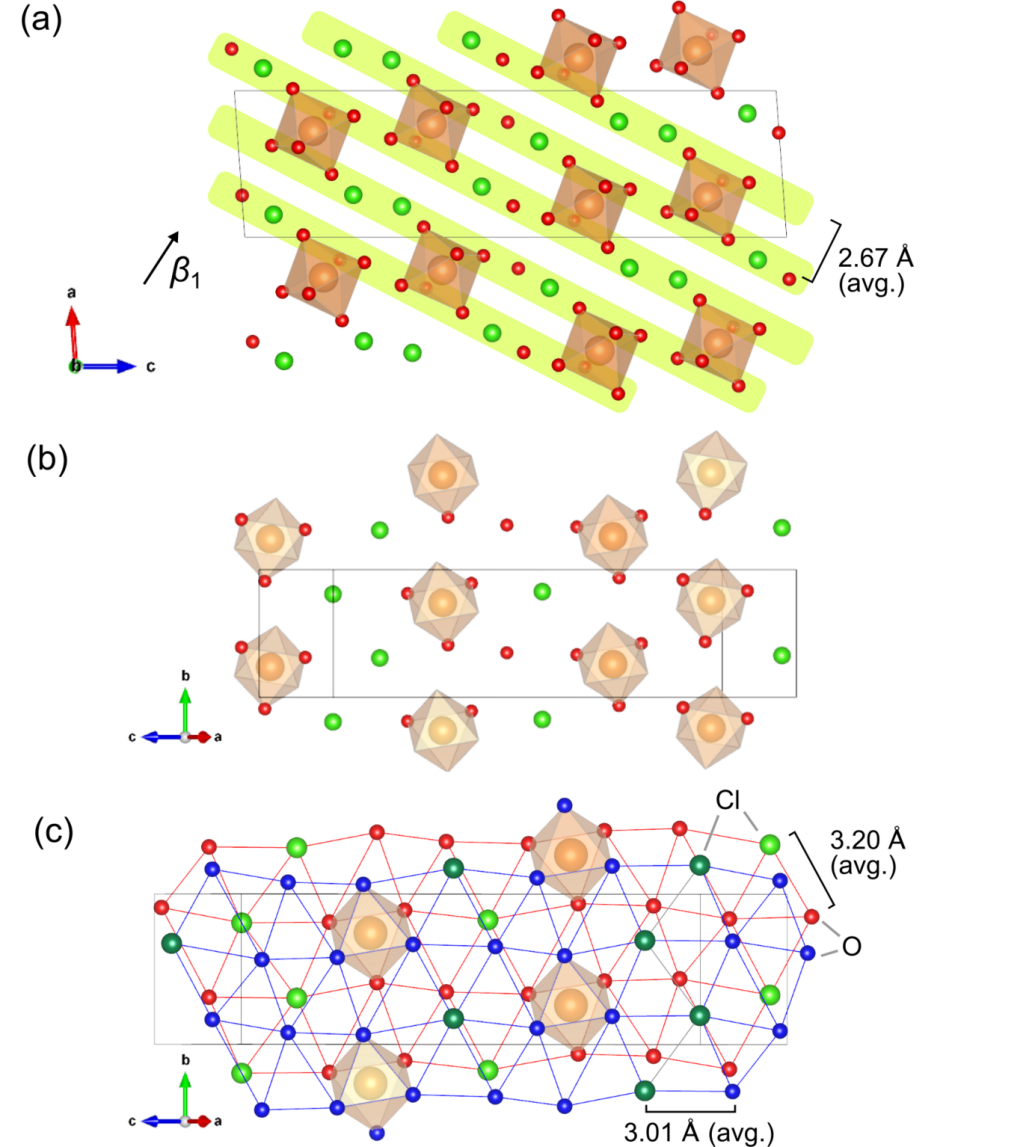
Extracted structure components in MgCl_2_·7H_2_O. (*a*) View along the *b* axis. Layers formed by Cl and O atoms, which are almost perpendicular to *n*_1_, are coloured yellow. (*b*) Extracted single layer specified in (*a*). (*c*) View along *n*_1_ direction. Red/blue, deep/light green and orange balls represent oxygen, chlorine, and magnesium atoms, respectively. Oxygen and chlorine atoms in different layers are distinguished by different colouring in (*c*). Atoms in the same layers are connected by red/blue lines. Hydrogen atoms are omitted for clarity. The inter/intralayer displacements between anion sites are described as average in the periodicity from the single-crystal X-ray diffraction at 2.5 GPa and 298 K for MgCl_2_·7H_2_O in (*a*) and (*c*).

The anisotropic compressibility of materials can be ascribed to either compressible structural units and/or their arrangements along *n*_1_ or less compressible units and/or their arrangement in a plane perpendicular to *n*_1_. For example, Fortes, Fernandez-Alonso, Tucker & Wood (2017 ▸) pointed out the connection between the compression behaviours of MgSO_4_·11D_2_O and the orientation of water hexadecamer connected by hydrogen bonds including bifurcated hydrogen bonds. Here, the magnitudes of β_2_ and β_3_ are almost identical in the pressure range of 2–4.5 GPa. Assuming incompressible structural features in these directions, we focus on a plane spanned by *n*_2_ and *n*_3_, which approximately corresponds to the (102) plane. Having a look at the cross-section on the *ac* plane, oxygen and chlorine are almost on a plane parallel to (102) within a deviation of 0.6 Å [Fig. 7 ▸(*a*)] with a hexagonal-like arrangement [Fig. 7 ▸(*b*)]. These layers stack along *n*_1_ with a slight shift [Fig. 7 ▸(*c*)]. The most compressible feature along *n*_1_ in MC7 would be attributed to the smaller free volumes within the layer and the relative ease of decreasing interlayer distances.

In contrast to the case of MgSO_4_·11D_2_O where the most compressible direction is parallel to bifurcated hydrogen bonds (Fortes, Fernandez-Alonso, Tucker & Wood, 2017 ▸), bifurcated hydrogen bonds in MC7d (D2*B*⋯Cl1) are perpendicular to the most compressible direction *n*_1_. Taking into account that the volume compressibilities are closer to anhydrous salts than water ice, the compression behaviours of MC7 are considered to be more dominated by the repulsions among atoms. In other words, the bifurcated bonds in MC7 would be the outcome of the hydrogen atoms escaping to a gap among other atoms against the compression. The linkage between the compression behaviours and apparent structural features such as bifurcated hydrogen bonds is not straightforward, especially between ambient- and high-pressure phases.

## 4. Concluding remarks

We identified magnesium chloride hydrate with odd hydration number, MgCl_2_·7H_2_O, (and MgCl_2_·7D_2_O) by *in*-*situ*X-ray and neutron diffraction. Its hydration number and crystal structure have been first determined by single-crystal X-ray diffraction, and the detailed structural features are elucidated by powder neutron diffraction and DFT calculations.

This hydrate is considered as the stable phase at least in the conditions of 323–353 K and 3–4 GPa compared to MgCl_2_·6H_2_O and MgCl_2_·10H_2_O (and their deuterated counter­parts). Further heating or compression will change the mutual stabilities. There is a possibility of unknown hydrates with lower hydration numbers, considering the dehydration behaviour from MgCl_2_·10H_2_O to MgCl_2_·7H_2_O as well as the cases of dehydration of MgCl_2_·6H_2_O upon heating at ambient pressure (Sugimoto *et al.*, 2007 ▸) and MgSO_4_·11H_2_O upon compression (Fortes, Fernandez-Alonso, Tucker & Wood, 2017 ▸; Fortes *et al.*, 2017 ▸).

The derived structure contains some unique structural features such as bifurcated hydrogen bonds, and direct hydrogen bonds between water molecules coordinating with magnesium. Furthermore, the comparison of the structures obtained by neutron diffraction and DFT calculation suggested the possibility of orientational disorder of water molecules. This potential orientational disorder requires reconnection of hydrogen bonds between water and chlorine, which differs from the case of water ice and a recently found salt hydrate, NaCl·13H_2_O (Yamashita *et al.*, 2023 ▸), of which orientational disorder can be examined through the graph-based approach. Complex packing of atoms to reduce the volume would induce the distorted structure compared to ambient pressure and lower-pressure phases of MgCl_2_ hydrates (Agron & Busing, 1985 ▸; Hennings *et al.*, 2013 ▸; Komatsu *et al.*, 2015 ▸; Yamashita *et al.*, 2019 ▸).

The salt-water system is still not fully explored in high-pressure regimes although their information is necessary from geological aspects to predict planetary dynamics and salt partitioning in icy bodies (Journaux *et al.*, 2017 ▸; Journaux *et al.*, 2023 ▸). To elucidate the phase relation, we first need to restrict the candidates of the constituents appearing in the salt-water system. We showed a new MgCl_2_ hydrate with a previously missing hydration number. The next step would be the detailed characterizations of the substances. The alcohol mixing approach enables us to obtain single crystals without the coexistence of other crystalline phases. This approach can be applied to further investigations that require single-crystalline samples, such as Brillouin scattering. This study will facilitate the hunting of unidentified hydrates in salt-water systems.

## Supplementary Material

Crystal structure: contains datablock(s) MC7_50256P21N_publ, MC7_50256P21N_overall, MC7_50256P21N_phase_1, MC7_50256P21N_phase_2, MC7_50256P21N_p_01, 1. DOI: 10.1107/S205252062400903X/ne5015sup1.cif

Structure factors: contains datablock(s) 1. DOI: 10.1107/S205252062400903X/ne5015sup2.hkl

Supporting information file. DOI: 10.1107/S205252062400903X/ne5015sup3.pdf

Supporting information file. DOI: 10.1107/S205252062400903X/ne50151sup4.cml

CCDC references: 2388640, 2388641, 2388642

## Figures and Tables

**Table 1:** Experimental details of structure refinements for MgCl_2_·7H_2_O from single-crystal X-ray diffraction and MgCl_2_·7D_2_O from powder neutron diffraction

	MgCl_2_·7H_2_O	MgCl_2_·7D_2_O
Crystal data		
Chemical formula	MgCl_2_(H_2_O)_7_	MgCl_2_(D_2_O)_7_
*M*_r_	221.32	235.40
Crystal system, space group	Monoclinic, *P*2_1_/*n*	Monoclinic, *P*2_1_/*n*
Pressure (GPa)	2.5	3.1
Temperature (K)	298	300
*a*, *b*, *c* (Å)	6.2669 (13), 5.6607 (12), 23.125 (5)	6.1815 (3), 5.62680 (17), 22.8254 (8)
β (°)	94.105 (10)	94.861 (3)
*V* (Å^3^)	818.3 (3)	791.06 (4)
*Z*	4	4
Radiation type	X-ray (Mo *K*α)	Spallation neutron
μ (mm^−1^)	0.86	–
Specimen shape, size (mm)	Platelet, 0.13 × 0.06 × 0.06	Back-to-back hemisphere, ϕ6
	
Data collection		
Diffractometer	Rigaku, R-AXIS IV^++^	PLANET (BL11), MLF, J-PARC
Specimen mounting	Diamond anvil cell with Boehler-Almax type anvils and PFA inner gasket	Pressure-temperature controlling system (the MITO system)
Scan method	ϕ scans for 80 frames by 1°	Time-of-flight
No. of measured, independent and observed [*I* > 2σ(*I*)] reflections	1190, 629, 627	–
*R*_int_	0.031	–
2θ_min_, 2θ_max_ values (°)	3.3, 32.2	78.7, 101.3
	
Refinement		
Computer program	*SHELXL2014* (Sheldrick, 2015 ▸)	*GSAS* (Larson & Von Dreele, 2004 ▸)
*R* factors and goodness of fit	*R*[*F*^2^ > 2σ(*F*^2^)] = 0.054, *wR*(*F*^2^) = 0.145, *S* = 1.22	*R*_p_ = 0.026, *R*_wp_ = 0.031, *R*_exp_ = 0.010, *R*(*F*^2^) = 0.1686, χ^2^ = 10.62
No. of parameters	41	108
No. of restraints	–	26
Δρ_max_, Δρ_min_ (e Å^−3^)	0.62, −0.32	–

**Table 2:** Fitted parameters of Murnaghan integrated linear equation of state (MILEOS; Murnaghan, 1944 ▸) for MgCl_2_·7H_2_O Parameters are derived using data from both compression and decompression. Fit parameters individually derived from each data of compression or decompression are in Table S3.

	*x*_0_	*K*_0_ (GPa)	*K*′
*a* (Å)	6.362 (10)	114 (11)	−2 (3)
*b* (Å)	5.92 (7)	17 (11)	41 (3)
*c* (Å)	24.1 (3)	25 (15)	27 (4)
β (°)	89 (41)	−3 (91)	−70 (26)
*V* (Å^3^)	879 (4)	21.4 (18)	5.5 (5)

**Table 3:** Derived linear and volume compressibilities by fitting linear expressions to the pressure dependences of the elastic strain tensor’s eigenvalues Parameters are derived using data from both compression and decompression. Volume compressibility is here defined as *n_V_* = *n*_1_ + *n*_2_ + *n*_3_.

	*n*_0_ (GPa^−1^)	*n*′
*n*_1_	1.82 × 10^−2^ (16)	−1.5 × 10^−3^ (4)
*n*_2_	1.34 × 10^−2^ (13)	−2.0 × 10^−3^ (3)
*n*_3_	6.9 × 10^−3^ (14)	−4 × 10^−4^ (4)
*n_V_*	3.8 × 10^−2^ (2)	−3.8 × 10^−3^ (6)
