# Supplementary material for: Crystal structure and compressibility of magnesium chloride heptahydrate found under high pressure
Source: Acta Crystallogr B Struct Sci Cryst Eng Mater. 2024 Oct 21;80(Pt 6):695–705. doi: 10.1107/S205252062400903X (PMC11789165; doi:10.1107/S205252062400903X)
Supplement: Supplementary file 3 [file b-80-00695-sup3.pdf]

STRUCTURAL SCIENCE
CRYSTAL ENGINEERING
MATERIALS

Volume 80 (2024)

Supporting information for article:

Crystal structure and compressibility of magnesium chloride heptahydrate by *in-situ* diffraction techniques under high pressure

Keishiro Yamashita, Kazuki Komatsu, Takanori Hattori, Shinichi Machida and Hiroyuki Kagi

S1. Preliminary powder x-ray diffraction

To confirm the phase behaviour of $MgCl_2{\cdot}10H_2O$ and the unknown phase ($MgCl_2{\cdot}7H_2O$ determined in this study), powder diffraction patterns were measured. A solution of $MgCl_2$:H_2O = 1:11 (mol) was loaded into the diamond anvil cell. A mixture of $MgCl_2{\cdot}10H_2O$ and ice VII crystals was obtained after compression to 4.2 GPa at 298 K (Figure S1a) as reported by Komatsu *et al.* (2015). The diffraction profiles changed after heating at 323 K for 2.5 hours (Figure S1b). The pressure in the sample space decreased by approximately 1 GPa after the phase transition. The pressure drop is larger than those expected for thermal expansion of DAC or pressure relaxation in the sample space, which are normally less than 0.3 GPa. After the heating, the diffraction peaks of $MgCl_2{\cdot}10H_2O$ disappeared whilst new peaks appeared along with the growth of diffraction peaks of ice VII, suggesting the dehydration of $MgCl_2{\cdot}10H_2O$ during the transformation. These new peaks are assigned to $MgCl_2{\cdot}7H_2O$ based on the structure model determined by the single-crystal x-ray diffraction. On the contrary, the mixture of $MgCl_2{\cdot}6H_2O$ and ice VII did not transform into $MgCl_2{\cdot}7H_2O$ even after a day at similar heating condition. The transition would be hindered kinetically since it requires the diffusion of water molecules and decomposition of stable pure ice.

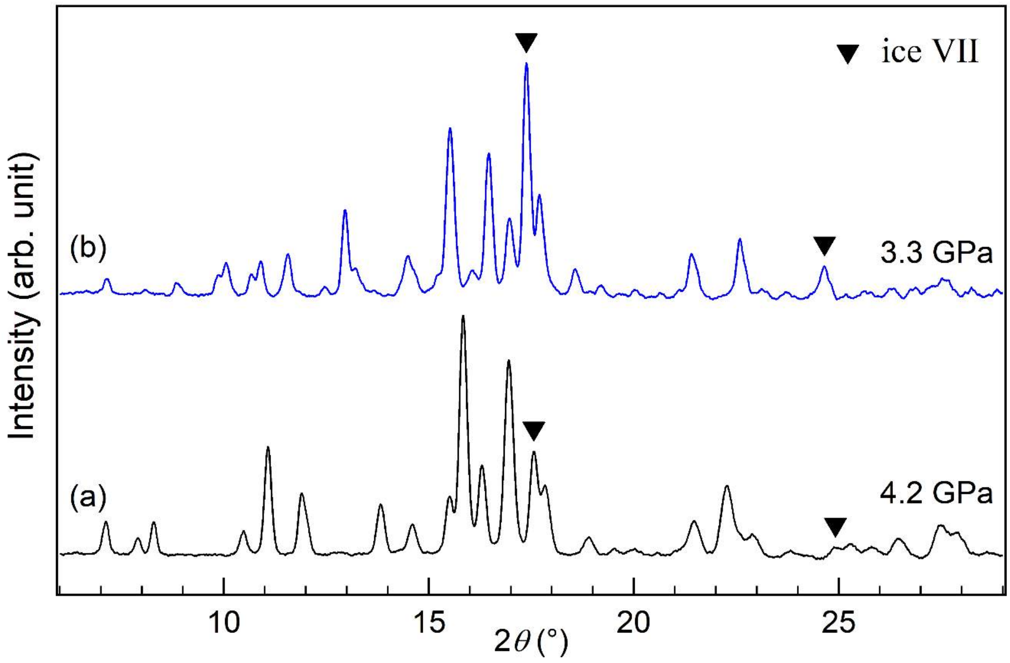

Figure S1 Representative diffraction patterns of (a) $MgCl_2{\cdot}10H_2O$ at 4.2 GPa and (b) $MgCl_2{\cdot}7H_2O$ at 3.3 GPa. The diffraction pattern (b) was measured after heating the sample shown in (a) at 323 K for 2.5 h. The initial sample solution was $MgCl_2$:H_2O = 1:11 (mol). Both diffraction profiles contain

peaks from a coexisting ice VII, marked with downward solid triangles. The diffraction patterns are measured for the same exposure time; the peak intensities can be compared to each other.

S2. High-pressure apparatus for single-crystal growth

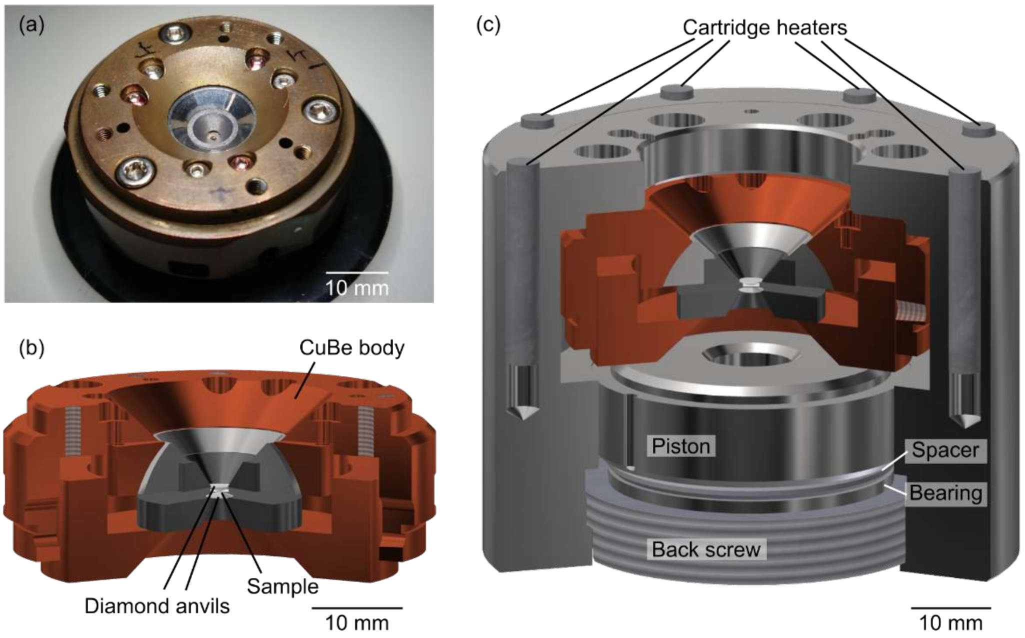

Figure S2 (a) Photograph of CuBe diamond anvil cell (DAC), (b) its schematic cutaway image, and (c) loading jacket equipped with heaters. Eight cartridge heaters can be loaded into the jacket. Four heaters were used in this study. Transmitted light comes from the bottom and the sample space is observed from the top using a microscope. Load is applied by the backscrew in the jacket using a gearbox.

S3. *In-situ* single-crystal growth in diamond anvil cells

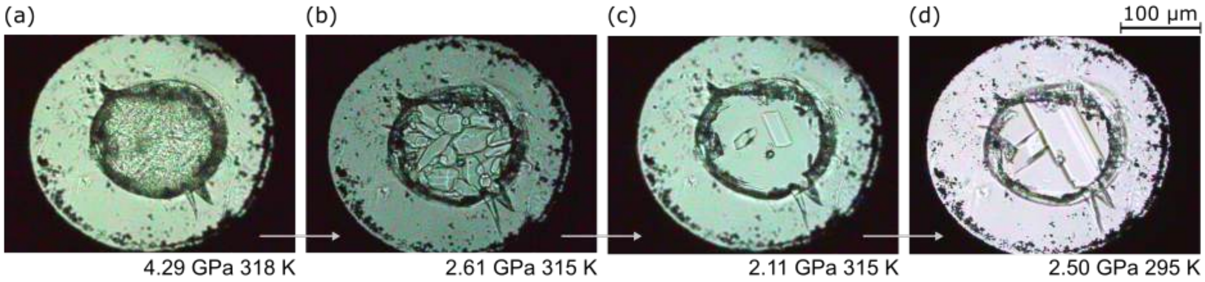

Figure S3 Photographs of the growth scheme of $MgCl_2 \cdot 7H_2O$ single crystals from the alcohol-mixed $MgCl_2$ aqueous solution in the diamond anvil cell. Transmitted light comes from the back side

of the photo (metal gaskets are seen as black shades around the PFA inner gasket). The diffraction from a larger crystal located on the right side in (d) was used for the structure refinement.

S4. Orientational disorder in $MgCl_2 \cdot 7D_2O$

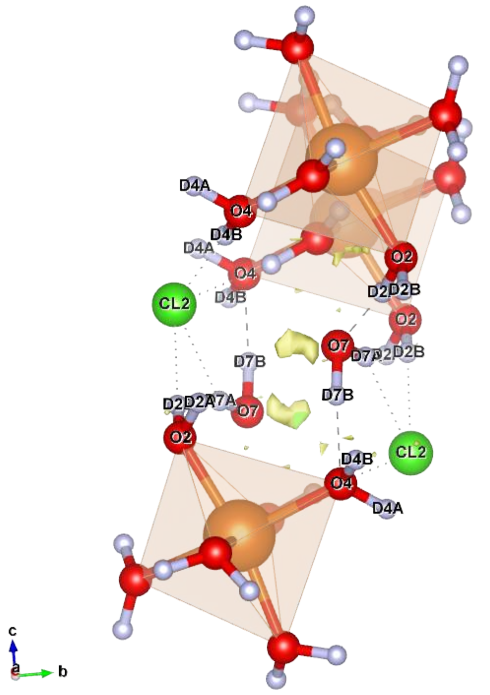

Figure S4 Difference Fourier map from neutron diffraction data using the DFT-optimised structure in configuration 1. The yellow clouds correspond to the positive residuals, suggesting the possibility of additional deuterium sites not included in the structure model.

Table S1 Initial configuration of 27 candidates of $MgCl_2 \cdot 7H_2O$ with $P2_1/n$ unit cell evaluated by the DFT calculation.

Occupancies for H7A-C, H6A-C, and H1A-C in each configuration are listed in rows 1–27. Rows 28–30 show the coordinates of these sites as their initial positions in the $P2_1/n$ cell setting.

	H7			H6			H1		
Configuration	A	B	C	A	B	C	A	B	C
1	1	1	0	1	1	0	1	1	0
2	1	0	1	1	1	0	1	1	0
3	0	1	1	1	1	0	1	1	0
4	1	1	0	1	0	1	1	1	0
5	1	0	1	1	0	1	1	1	0
6	0	1	1	1	0	1	1	1	0
7	1	1	0	0	1	1	1	1	0
8	1	0	1	0	1	1	1	1	0
9	0	1	1	0	1	1	1	1	0
10	1	1	0	1	1	0	1	0	1
11	1	0	1	1	1	0	1	0	1
12	0	1	1	1	1	0	1	0	1
13	1	1	0	1	0	1	1	0	1
14	1	0	1	1	0	1	1	0	1
15	0	1	1	1	0	1	1	0	1
16	1	1	0	0	1	1	1	0	1
17	1	0	1	0	1	1	1	0	1
18	0	1	1	0	1	1	1	0	1
19	1	1	0	1	1	0	0	1	1
20	1	0	1	1	1	0	0	1	1
21	0	1	1	1	1	0	0	1	1
22	1	1	0	1	0	1	0	1	1
23	1	0	1	1	0	1	0	1	1
24	0	1	1	1	0	1	0	1	1
25	1	1	0	0	1	1	0	1	1
26	1	0	1	0	1	1	0	1	1
27	0	1	1	0	1	1	0	1	1
x	0.651	0.828	0.720	0.539	0.532	0.700	0.352	0.449	0.450
y	0.732	0.630	0.500	0.930	0.903	0.903	0.308	0.535	0.260

	z	0.50<br>5	0.46<br>7	0.51<br>5	0.37<br>9	0.31<br>1	0.31<br>1	0.37<br>7	0.41<br>0	0.41<br>0

S5. Isothermal compressibility of $MgCl_2{\cdot}7H_2O$ from synchrotron x-ray diffraction

Table S2 Lattice parameters of $MgCl_2{\cdot}7H_2O$ in $P2_1/n$ unit cell obtained by powder synchrotron x-ray diffraction at 298 K.

	p (GPa)	*a* (Å)	*b* (Å)	*c* (Å)	*β* (°)	*V* ($Å^3$)
Compression	2.491	6.2214 (3)	5.6492 (2)	22.9558 (7)	94.644 (4)	804.15 (3)
	2.588	6.2144 (2)	5.64359 (12)	22.9326 (4)	94.681 (2)	801.60 (2)
	2.693	6.2093 (2)	5.63867 (12)	22.9047 (4)	94.737 (2)	799.21 (2)
	2.996	6.1927 (2)	5.62703 (11)	22.8412 (4)	94.860 (2)	793.07 (2)
	3.202	6.1813 (2)	5.61884 (13)	22.8004 (4)	94.936 (2)	788.96 (3)
	3.454	6.1664 (2)	5.60950 (15)	22.7510 (5)	95.027 (3)	783.95 (3)
	3.712	6.1503 (3)	5.60039 (19)	22.7018 (7)	95.122 (3)	778.82 (4)
	3.807	6.1442 (3)	5.5971 (2)	22.6835 (7)	95.156 (3)	776.93 (4)
	4.006	6.1311 (4)	5.5911 (3)	22.6515 (10)	95.238 (4)	773.24 (5)
	4.397	6.1095 (5)	5.5801 (3)	22.5916 (13)	95.355 (5)	766.83 (7)
	4.475	6.1043 (5)	5.5779 (3)	22.5753 (13)	95.390 (6)	765.27 (7)
	4.849	6.0872 (5)	5.5683 (3)	22.5230 (15)	95.489 (6)	759.93 (7)
Decompression	4.809	6.0884 (5)	5.5685 (3)	22.5144 (13)	95.539 (5)	759.75 (6)
	4.745	6.0928 (4)	5.5691 (3)	22.5214 (12)	95.512 (5)	760.64 (6)
	4.709	6.0949 (4)	5.5700 (3)	22.5257 (12)	95.496 (5)	761.21 (6)
	4.546	6.1057 (4)	5.5751 (3)	22.5492 (11)	95.472 (5)	764.07 (6)
	4.389	6.1142 (4)	5.5809 (3)	22.5780 (11)	95.455 (4)	766.93 (5)
	4.207	6.1237 (4)	5.5865 (2)	22.6091 (10)	95.416 (4)	770.01 (5)
	3.907	6.1424 (3)	5.5955 (2)	22.6601 (9)	95.324 (4)	775.46 (5)
	3.801	6.1488 (3)	5.5973 (2)	22.6745 (8)	95.263 (4)	777.10 (4)
	3.501	6.1665 (3)	5.6066 (2)	22.7246 (6)	95.147 (3)	782.50 (4)
	3.195	6.1841 (2)	5.6178 (2)	22.7805 (6)	95.034 (3)	788.36 (3)
	2.857	6.2026 (2)	5.6316 (1)	22.8587 (5)	94.849 (3)	795.61 (3)
	2.692	6.2107 (2)	5.6392 (1)	22.9029 (5)	94.750 (3)	799.38 (2)

Lattice parameters and their uncertainties were derived by the Rietveld analysis using the *GSAS* software (Larson & Dreele, 2004). Pressure values were derived from the lattice parameter of coexisting ice VII (Klotz *et al.*, 2017).

Table S3 Individual fit parameters of Murnaghan integrated linear equation of state (MILEOS; equation (1); (Murnaghan, 1944)) for $MgCl_2·7H_2O$ upon compression and decompression.

	x_0*	K_0 (GPa)	*K*'
Compression			
a	6.373 (12)	102 (12)	1 (3)
b	6.03 (15)	6 (8)	45 (2)
c	24.06 (9)	26 (5)	27.5 (14)
β	93.09 (15)	−101 (18)	−45 (2)
V	883 (5)	20 (2)	6 (7)
Decompression			
a	6.350 (5)	131 (7)	−6.6 (17)
b	5.84 (6)	37 (26)	36 (7)
c	24.3 (5)	16 (15)	30 (4)
β	85 (1578)	0 (113)	−72 (31)
V	874 (5)	24 (3)	4.8 (7)

Errors are estimated from the fitting of data points. *The units are Å for *a*, *b*, and *c*-axes, ° for *β*, and $Å^3$ for *V*.
